# Feasibility of vancomycin AUC_24_ monitoring using peak and trough concentrations in pediatric patients: a prospective multicenter study

**DOI:** 10.3389/fphar.2026.1790042

**Published:** 2026-04-07

**Authors:** Majed H. Nahari, Abdullah Alsultan, Renad A. Alshuraim, Abdullah Altuwayjiri, Sultan Alotaibi, Majed Aljeraisy

**Affiliations:** 1 Pharmaceutical Care Services, King Abdullah bin Abdulaziz University Hospital, Riyadh, Saudi Arabia; 2 Collage of Pharmacy, Princess Nourah bint Abdulrahman University, Riyadh, Saudi Arabia; 3 Department of Clinical Pharmacy, College of Pharmacy, King Saud University, Riyadh, Saudi Arabia; 4 Pharmaceutical Care Services, King Saud Medical City, Riyadh, Saudi Arabia; 5 Pharmaceutical Care Services, King Salman Specialized Hospital, Taif, Saudi Arabia; 6 King Abdullah International Medical Research Center, Riyadh, Saudi Arabia; 7 College of Pharmacy, King Saud bin Abdulaziz University for Health Science, Riyadh, Saudi Arabia

**Keywords:** area under the curve (AUC), AUC-guided dosing, feasibility study, pediatric pharmacokinetics, therapeutic drug monitoring, vancomycin

## Abstract

**Background:**

Vancomycin remains a key medication in treating infections in pediatric patients, particularly those caused by methicillin-resistant *Staphylococcus aureus* (MRSA). Conventional trough-based monitoring has shown limited accuracy in predicting therapeutic exposure and nephrotoxicity. Recent guidelines recommend area under the concentration–time curve (AUC)-based monitoring, but pediatric evidence remains scarce. Our objective is to assess the feasibility of vancomycin AUC_24_ monitoring using peak–trough concentrations in routine pediatric clinical practice and examine its correlation with the trough levels.

**Methods:**

We conducted a prospective, multicenter study in 70 pediatric inpatients receiving vancomycin at two tertiary hospitals in Riyadh, Saudi Arabia. Vancomycin AUC_24_ was calculated using first-order pharmacokinetics from peak and trough levels. AUC_24_ was compared with the trough concentrations and dosing regimens.

**Results:**

Implementation of AUC_24_-based monitoring was feasible in routine pediatric workflow. Pharmacists successfully calculated AUC_24_ using the Sanford Guide mobile app, with minimal training required for nurses on sampling. The correlation between the trough levels and AUC_24_ was strong (ρ = 0.789, p < 0.001), but the trough values did not consistently predict target attainment (AUC_24_ 400 μg h/mL–600 μg h/mL). At trough concentrations of 10 μg/mL–15 μg/mL, only approximately half of the patients achieved the target AUC_24_ range (400 μg h/mL–600 μg h/mL), whereas at troughs of 15 μg/mL–20 μg/mL, nearly half of the patients exceeded the safe exposure thresholds, underscoring the limitations of trough-based monitoring.

**Conclusion:**

AUC_24_ estimation from peak and trough samples was feasible in routine pediatric workflow, providing more accurate exposure assessment than trough-guided monitoring. Implementation may improve efficacy and minimize nephrotoxicity.

## Introduction

Vancomycin is an important glycopeptide antibiotic extensively utilized in pediatric clinical settings, especially for severe Gram-positive infections, including those caused by MRSA (Patel et al., 2024; Siddiqui and Koirala, 2018). Methicillin-resistant *Staphylococcus aureus* (MRSA) remains a significant pathogen in complex infections, affecting pediatric patients with conditions such as bacteremia, endocarditis, osteomyelitis, pneumonia, septic arthritis, meningitis, and skin and soft tissue infections (Lodise et al., 2014). Invasive MRSA infections in children lead to considerable health challenges, such as extended hospitalizations, severe complications, and a heightened requirement for intensive-care assistance (Sun et al., 2023). Although the rates of mortality in children are less than those seen in adults, it continues to hold significant clinical relevance: extensive cohort studies indicate that the overall mortality rates of bloodstream infections caused by MRSA are approximately 1%–2%, with complications arising in nearly a quarter of the cases (Regen et al., 2019). In pediatric cohorts with *Staphylococcus aureus* sepsis, the reported mortality rates are lower than those in adults, generally ranging from approximately 3% to 6%. However, higher rates of adverse clinical outcomes have been reported, including persistent bacteremia beyond 48 h and clinical failure—defined as recurrent bacteremia or mortality—in up to approximately 17% of cases (Lee et al., 2024). The results underscore the significant clinical impact of MRSA, emphasizing the critical role of vancomycin in pediatric antimicrobial treatment and the necessity of fine-tuning dosing to achieve an optimal balance between effectiveness and safety (Liu et al., 2011).

The clinical efficacy of vancomycin is primarily connected with a PK/PD parameter—the area under the concentration–time curve (AUC)/ minimum inhibitory concentration (MIC) ratio. Currently, an AUC/MIC ratio of ≥ 400 is generally considered the optimal target for predicting better clinical outcomes and reduced resistance development (Rybak et al., 2020). The Infectious Diseases Society of America (IDSA) 2011 guidelines initially recommended that vancomycin trough levels of 15 μg/mL–20 μg/mL be targeted for deep-seated infections to ensure attaining this therapeutic PK/PD target (Liu et al., 2011). Nevertheless, these guidelines were derived largely from adult data, and questions have been raised about their suitability and safety in children due to the differing physiological characteristics and unique pharmacokinetic traits in children (Alsultan et al., 2020). Targeting high trough concentrations (15 μg/mL–20 μg/mL) in children carries a substantial risk of overdosing and nephrotoxicity, as their pharmacokinetics differ significantly from those of adults (Rybak et al., 2020). The new IDSA treatment guidelines recommend AUC calculation (Bayesian one-sample or two-sample first-order calculation) instead of trough-guided monitoring. Some have suggested targeting trough concentrations but at the range of 10 μg/mL–15 μg/mL to avoid the risk of nephrotoxicity (Pham, 2020; Kishk et al., 2017).

More recently, pediatric studies have also challenged the practice of relying on high trough concentration as a surrogate marker for AUC/MIC attainment (Tkachuk et al., 2018). In a pediatric retrospective study, Kishk et al. (2017) showed that by using 15 mg/kg of vancomycin every 6 h, they achieved AUC/MIC ≥400 that corresponded to a trough ≈11 mg/L (median 11.4 mg/L); that is, it was markedly lower than the traditional 15 mg/L–20 mg/L target in adults (Kishk et al., 2017). Likewise, in another study, Alsultan et al. (2020) showed that even infants and young children achieved therapeutic AUC/MIC targets at vancomycin trough concentrations below the previously recommended target range (Alsultan et al., 2020). Another systematic review corroborated these results and recommended that targeting trough levels of 6 mg/L–10 mg/L would optimize AUC/MIC targets in pediatric patients (Tkachuk et al., 2018).

In addition, several reports have correlated higher troughs, particularly 15 μg/mL–20 μg/mL, with an increased risk of nephrotoxicity in children. In a clinical environment, nephrotoxicity is a major issue that is responsible for adverse events in patients, as well as healthcare cost. In a retrospective review of 680 pediatric patients, a positive association was found between the risk of nephrotoxicity and higher vancomycin exposures, namely, estimated AUC_24_ of ≥ 800 mg h/L and trough concentrations of ≥ 15 μg/mL (Le et al., 2015). Other meta-analyses and studies also support this association of increased trough concentrations with the risk for nephrotoxicity in children (Woldu and Guglielmo, 2018; Fiorito et al., 2018). These data show that the main reason for using AUC-guided vancomycin doses in clinical settings is to make it safer and not to make it more effective. Using AUC-based monitoring, we can find pediatric patients who receive enough pharmacodynamic exposure while keeping their trough concentrations lower than what would normally be considered sub-therapeutic with trough-based monitoring. This means that we do not have to increase the dose, and the risk of nephrotoxicity decreases.

As evidence for AUC-based monitoring has increased and the constraints of trough monitoring have become more widely recognized, recent consensus guidelines from the American Society of Health-System Pharmacists (ASHP), IDSA, the Society of Infectious Diseases Pharmacists (SIDP), and the Pediatric Infectious Diseases Society (PIDS) now recommend AUC-based therapeutic drug monitoring (TDM) for pediatric patients receiving vancomycin (Rybak et al., 2020). This approach is expected to allow more precise individual dosing, minimize the potential nephrotoxicity risk, and optimize patient outcomes by targeting the PK/PD parameters that have been demonstrated to predict efficacy (Oda et al., 2020; Schouwenburg et al., 2025).

However, the experience in practice of using vancomycin AUC-based TDM to guide the dosage in a pediatric setting is minimally well documented. The practical consequences, ease of application, and clinical advantage of a transition from traditional trough-based monitoring to AUC-based monitoring in pediatrics are yet to be concluded. Accordingly, in this study, we aimed to evaluate the feasibility of implementing vancomycin AUC_24_ monitoring using peak and trough concentrations in pediatric inpatients and to examine its correlation with traditional trough-based monitoring.

## Objectives

The primary objective of this study was to evaluate the feasibility of implementing vancomycin AUC_24_ monitoring using peak and trough concentrations in routine pediatric clinical practice. A secondary objective was to examine the relationship among AUC_24_, trough concentrations, and dosing regimens.

As a secondary objective, we aimed to assess the correlation between the initial AUC_24_ and vancomycin trough concentrations.

## Methods

This prospective study was designed to assess the feasibility of individualizing vancomycin dosing to achieve AUC_24_ targets based on the simplified two-equation-based strategy using peak and trough serum concentrations in pediatric patients (Pai et al., 2014; Alsultan et al., 2020). It was conducted as a multicenter study among inpatient children at King Abdullah Specialist Children’s Hospital (KASCH) and King Saud Medical City (KSMC) in Riyadh, Saudi Arabia. The study included pediatric patients aged between 1 month and 14 years who received optimal vancomycin for proven or suspected infections for more than 48 h. Patients were excluded if they had acute or chronic kidney disease, if they were critically ill, or if parental consent was declined. Patients with vancomycin peak or trough concentrations outside the predefined optimal sampling windows were excluded to ensure accurate AUC_24_ estimation using first-order pharmacokinetic calculations.

## Data collection procedure

The study was approved by the ethics committees at King Abdullah International Medical Research Center and King Saud Medical City. Written informed consent from the parents or legal guardian was obtained before commencing prospective data collection. The vancomycin AUC_24_ was calculated using vancomycin peak and trough serum concentrations and first-order equations (simplified two-equation strategy, Pia et al.), with the peak and trough representing the maximum and minimum serum concentration levels, respectively.

- Trough level: collected within 30 min before administering the fourth dose.
- Peak level: collected at least 1 h after completing the infusion of the third dose.

For all calculations, a vancomycin MIC of 1 μg/mL was assumed, which is consistent with the most recent hospital antibiogram. The Sanford Guide mobile application was used for AUC estimation; although validated in adult cohorts, its use in pediatric populations has had limited prior validation, which was considered when interpreting results. The pharmacist time required to perform vancomycin AUC_24_ calculations and related documentation was not formally quantified in this study.

### AUC_24_ calculation

The following standardized steps were used to calculate the vancomycin AUC_24_. All calculations were performed using the *Sanford Guide to Antimicrobial Therapy* mobile application, which incorporates validated vancomycin AUC calculator functions.

1. Elimination rate constant (ke): calculated using the formula ke = Ln(C1/C2)/t.
2. True peak concentration: calculated as true peak = peak/e(-ke) (T1 - Tinf).
3. True trough concentration: calculated as true trough = trough × e(-ke) (Tau - T2).
4. AUC infusion (AUC info): calculated as AUC info = (true trough + true peak/2) × Tinf.
5. AUC elimination (AUC elim): calculated as AUC elim = (true peak - true trough)/ke.
6. Total AUC24: calculated as AUC24 = (AUC info + AUC elim) × (24/Tau).

### Statistical analysis

Descriptive statistics were reported as medians with interquartile ranges (IQRs). Spearman correlation coefficients were used to assess associations among AUC_24_, trough levels, and total daily doses (mg/kg/day). A p-value <0.05 was considered statistically significant. Statistical analysis was performed using the SPSS software version 26.0 (IBM, Chicago, IL, United States).

### Ethical considerations

The study was approved by the Institutional Research Bioethics Committee at King Abdullah International Medical Research Center (KAIMRC) IRB (NRC21R/140/04) and King Saud Medical City (KSMC) IRB (H-01-R-053). Consent from parents or legal guardians were obtained before data were collected. Patient privacy and confidentiality was always upheld, and data  were stored securely in the hospital facilities.

## Results

Implementation of the AUC_24_-based approach was feasible in our setting, with baseline characteristics summarized in Table 1. AUC_24_ calculations were completed by clinical pharmacists as part of routine clinical duties using the Sanford Guide mobile application, without the need for dedicated personnel or workflow restructuring. Pharmacists were able to perform these calculations efficiently within standard practice, and only brief training was required for the nursing staff to ensure appropriate timing of sample collection.

**TABLE 1:** Baseline characteristics (N = 70).

Age (years), median (IQR)	3.15 (0.93–7.93)
Age-group, n (%)	
Infant (1 month to <1 year)	50 (71.43)
Children (≥1 year to 14 years)	20 (28.57)
Gender, n (%)	
Male	40 (57.14)
Female	30 (42.86)
Height (cm), median (IQR)	93 (63.25–120)
Weight (kg), median (IQR)	12.63 (6.23–22.3)
Dose (mg/kg/dose), median (IQR)	15 (13–15)
Dose (mg/kg/day), median (IQR)	59.15 (48.38–60)
Frequency, median	4
Trough level (ug/mL), median (IQR)	8.19 (4.97–12.57)
Peak level (ug/mL), median (IQR)	20.08 (14.89–26.42)
Elimination rate constant (Ke hr^-1^), median (IQR)	0.34 (0.25–0.42)
Half-life, median (IQR)	2.06 (1.67–2.88)
VD (L/kg), median (IQR)	1.27 (0.95–1.71)
AUC_24_ (ug/mL * hr), median (IQR)	338.5 (262.5–523.5)

The correlation analyses showed a statistically strong positive correlation between vancomycin trough levels and AUC24 values (ρ = 0.789, p < 0.001) (Figure 3), indicating that higher trough levels were associated with higher drug exposure quantified by AUC_24_.

**FIGURE 1:**
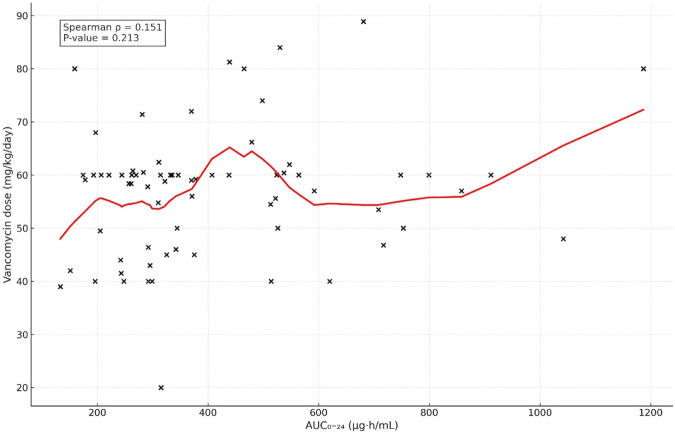
Correlation between AUC_0–24_ and vancomycin dose.

On the other hand, a poor correlation was found between vancomycin dose (mg/kg/day) and AUC_24_ (ρ = 0.151, p = 0.213). Figure 1 implies that the dose alone had little predictive power for the accurate value of AUC. There was also no correlation between vancomycin dose and trough concentration (ρ = 0.023, p = 0.853), highlighting the inability to rely on trough concentration or dose as a means to predict appropriate drug exposure in pediatric patients.

**FIGURE 2:**
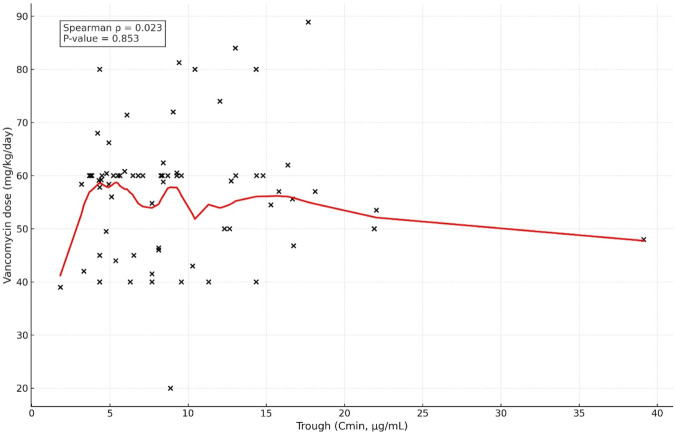
Correlation between trough level (Cmin) and vancomycin dose.

We observed that at a trough level 10–15 μg/mL, only ∼50% of patients achieved the target, with the distribution of AUC attainment across trough ranges detailed in Table 2 and at 15 μg/mL–20 μg/mL, nearly half of the patients exceeded safe exposure thresholds. No particular trough concentration range consistently ensured the desired AUC_24_ (400 μg h/mL–600 μg h/mL). Although trough concentrations spanning 10 μg/mL–15 μg/mL were associated with the highest proportion of patients within the therapeutic AUC range (approximately 50%), substantial variability remained. These findings highlight the difficulty in accurately predicting AUC_24_ based solely on trough concentrations. Therefore, in circumstances where AUC-based monitoring is unfeasible, targeting trough concentrations of 10 μg/mL–15 μg/mL might provide a pragmatic approach to reduce the likelihood of overexposure and potential nephrotoxicity. This confirms the poor correlation between the vancomycin dose and trough levels, as mentioned before (Figure 2). This is consistent with prior studies showing that at trough levels of 15 μg/mL–20 μg/mL, there is a risk of high AUCs and, consequently, nephrotoxicity, whereas at 10 μg/mL–15 μg/mL, approximately 60% of the patients achieved the target AUC.

**TABLE 2:** Proportion of patients who achieved an AUC 400 μg h/mL–600 μg h/mL.

Trough range	Within AUC 400–600, n (%)	Below, n (%)	Above, n (%)
<10 (n = 47)	6 (13)	39 (83)	2 (4.3)
10–15 (n = 13)	6 (46)	4 (31)	3 (23)
15–20 (n = 7)	4 (57)	0	3 (43)
>20 (n = 3)	0	0	3 (100)

**FIGURE 3:**
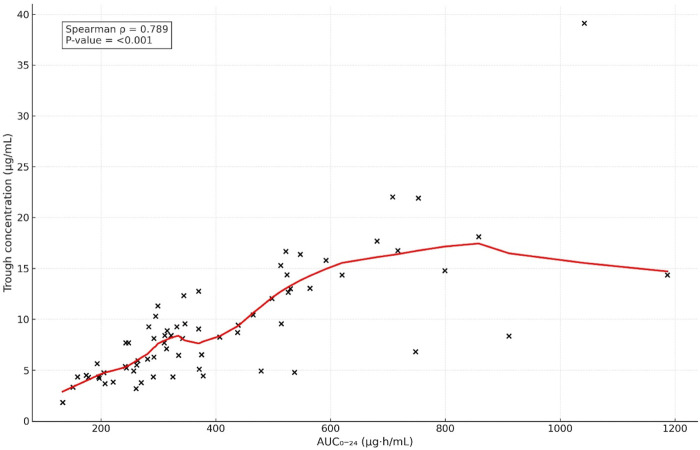
Correlation between AUC_0–24_ and trough level (Cmin).

## Discussion

While the observed relationships among the vancomycin dose, trough concentrations, and AUC_24_ have been previously described, their inclusion in this study serves to confirm these patterns within a prospective, real-world pediatric implementation setting and to support the feasibility of AUC_24_-guided monitoring rather than to establish novel pharmacokinetic associations.

Our results demonstrated a statistically significant correlation between vancomycin trough concentrations and AUC_24_; however, this relationship did not reliably predict the attainment of therapeutic AUC targets, as substantial variability was observed across all trough ranges. These findings are consistent with previously published pediatric pharmacokinetic studies and serve to contextualize our results within existing literature. Kishk et al. and Tkachuk et al. demonstrated that a wide range of AUC values may correspond to the same trough concentration, leading to both sub-therapeutic exposure and excessive dosing when trough-based monitoring is used alone. This variability highlights the insufficiency of trough monitoring as an isolated technique and supports AUC-guided monitoring as a more accurate and therapeutically pertinent method. In our group, the median AUC_24_ was 338.5 μg h/mL, indicating that therapeutic targets (AUC_24_ ≥ 400 μg h/mL) may not be reliably attained by conventional dosage and trough-based monitoring. These findings together support contemporary pediatric research that promotes individualized AUC-based dose optimization and further discourage reliance solely on trough concentrations for vancomycin TDM.

In contrast to the strong relationship evident between the trough levels and AUC_24_, weak and inconsistent associations between vancomycin doses (mg/kg/day) and AUC_24_ (r = 0.202, p = 0.094) demonstrate that dose alone is a poor predictor of exposure that achieves a therapeutic target. Similar findings have consistently been reported in pediatric pharmacokinetic studies demonstrating that standard weight-based dosing alone is an unreliable predictor of vancomycin exposure due to substantial interpatient variability (Barker et al., 2018; Murphy et al., 2021; Jung et al., 2024; Kanazawa et al., 2024; Meng et al., 2019; Vali et al., 2021) because of substantial inter-patient variability in clearance and the volume of distribution, particularly across different pediatric age-groups and developmental stages (Kishk et al., 2017). This finding reinforces existing evidence that dose-driven or trough-based strategies alone may inadequately predict vancomycin exposure in pediatric patients.

Furthermore, our data showed no association of vancomycin dose with trough concentrations (r = 0.027, p = 0.823), which supports literature highlighting under- or over-dosing risks when dosing is solely based on trough measurements (Tkachuk et al., 2018; Rybak et al., 2020).

On the basis of clinical relevance, the possibility of estimating vancomycin AUC_24_ based on the routine available serum concentration data reflects a pragmatic move toward a pharmacodynamic-oriented approach in pediatric vancomycin dosing. This strategy is consistent with updated consensus guidelines from ASHP, IDSA, SIDP, and PIDS, which recommend AUC-guided TDM for reducing the risk of toxicity and achieving optimal efficacy (Finch et al., 2017; Neely et al., 2018).

Our study has some limitations; the small sample size and the use of convenience sampling could limit the generalizability of the findings. In addition, patients with severe conditions or renal insufficiency were excluded, which might limit the applicability of the results on high-risk patients. This study did not assess patient-level clinical outcomes such as efficacy or nephrotoxicity; therefore, conclusions regarding the clinical superiority of AUC-based monitoring cannot be drawn.

Large, randomized, more diverse populations and prospective studies are needed in the future to determine the accuracy of our findings. Additionally, assuming a uniform MIC of 1 μg/mL may not accurately reflect the clinical variability across isolates, which could influence the generalizability of our AUC/MIC findings. Furthermore, we did not intend to link this correlation with either efficacy or toxicity as we did not assess any parameters related to both these outcomes.

## Conclusion

The results of this study support the utility and clinical value of estimating vancomycin AUC_24_ based on the peak and trough serum concentrations in pediatric patients. AUC-guided monitoring is a more accurate way to determine doses and avoids the shortcomings of trough-based strategies. Our results advocate for the incorporation of AUC-based TDM into standard pediatric clinical practice to promote the safe and effective use of vancomycin. By demonstrating feasibility in routine practice, in this study, we provide real-world evidence that AUC_24_ estimation can be integrated into pediatric clinical workflows with minimal disruption, thus enhancing both safety and efficacy. The wider application of this approach, together with additional research in different clinical settings, could be instrumental in harmonizing the use of the best possible dosing strategies of antibiotics for children with infections.

## Data Availability

The original contributions presented in the study are included in the article/supplementary material; further inquiries can be directed to the corresponding author.

## References

[B1] Alsultan A. Abouelkheir M. Albassam A. Alharbi E. Assiri A. Alqahtani S. (2020). AUC- vs trough-guided monitoring of vancomycin in infants. Indian J. Pediatr. 87 (5), 359–364. 10.1007/s12098-019-03162-5 31984471

[B2] Barker C. I. Standing J. F. Kelly L. E. Faught L. H. Needham A. C. Rieder M. J. (2018). Pharmacokinetic studies in children: recommendations for practice and research. Arch. Dis. Child. 103 (7), 695–702. 10.1136/archdischild-2017-314506 29674514 PMC6047150

[B4] Finch N. A. Zasowski E. J. Murray K. P. Mynatt R. P. Zhao J. J. Yost R. (2017). Impact of AUC-guided dosing on vancomycin-associated nephrotoxicity: a quasi-experiment. Antimicrob. Agents Chemother. 61 (12), e01293 10.1128/AAC.01293-17 28923869 PMC5700348

[B5] Fiorito T. M. Luther M. K. Dennehy P. H. LaPlante K. L. Matson K. L. (2018). Nephrotoxicity with vancomycin in the pediatric population: a systematic review and meta-analysis. Pediatr. Infect. Dis. J. 37 (7), 654–661. 10.1097/INF.0000000000001882 29280786

[B6] Jung D. Kishk O. A. Bhutta A. T. Cummings G. E. El Sahly H. M. Virk M. K. (2024). Evaluation of vancomycin dose needed to achieve 24-hour area under the concentration-time curve to minimum inhibitory concentration ratio greater than or equal to 400 using pharmacometric approaches in pediatric intensive care patients. Crit. Care Explor. 6 (10), e1159. 10.1097/CCE.0000000000001159 39352409 PMC11446596

[B7] Kanazawa N. Shigemi A. Amadatsu N. Arimura K. Shimono S. Oda K. (2024). A cohort study of the risk factors and the target AUC to avoid vancomycin-associated acute kidney injury in pediatric patients. J. Infect. Chemother. 30 (4), 323–328. 10.1016/j.jiac.2023.12.021 37940038

[B8] Kishk O. A. Lardieri A. B. Heil E. L. Morgan J. A. (2017). Vancomycin AUC/MIC and corresponding troughs in a pediatric population. J. Pediatr. Pharmacol. Ther. 22 (1), 41–47. 10.5863/1551-6776-22.1.41 28337080 PMC5341531

[B9] Le J. Ny P. Capparelli E. Lane J. Ngu B. Muus R. (2015). Pharmacodynamic characteristics of nephrotoxicity associated with vancomycin in children. J. Pediatr. Infect. Dis. Soc. 4 (4), e109–e116. 10.1093/jpids/piu110 26582878 PMC4681388

[B10] Lee Y. Kim G. Lee J. (2024). Optimal vancomycin AUC target in pediatric MRSA bacteremia: a Bayesian-guided approach. Pediatr. Infect. Dis. J. 10.1097/INF.0000000000004307 PMC12422622 40570348

[B11] Liu C. Bayer A. Cosgrove S. E. Daum R. S. Fridkin S. K. Gorwitz R. J. (2011). Clinical practice guidelines by the IDSA for the treatment of MRSA infections in adults and children. Clin. Infect. Dis. 52 (3), e18–e55. 10.1093/cid/ciq146 21208910

[B12] Lodise T. P. Drusano G. L. Zasowski E. Dihmess A. Lazariu V. Cosler L. (2014). Vancomycin exposure in patients with MRSA bloodstream infections: how much is enough? Clin. Infect. Dis. 59 (5), 666–675. 10.1093/cid/ciu398 24867791

[B13] Meng L. Wong T. Huang S. Mui E. Nguyen V. Espinosa G. (2019). Conversion from trough-guided to AUC-guided vancomycin dosing using two sample measurements in adults: implementation at an academic medical center. Pharmacotherapy 39 (4), 433–442. 10.1002/phar.2234 30739349

[B126] Murphy M. E. Tang Girdwood S. Goldman J. L. Scheetz M. H. Downes K. J. (2021). Precision dosing of vancomycin: in defence of AUC-guided therapy in children. J. Antimicrob. Chemother. 76 (10), 2494–2497. 10.1093/jac/dkab242 34096598 PMC8633448

[B26] Neely M. N. Youn G. Jones B. Jelliffe R. W. Drusano G. L. Rodvold K. A. (2018). Are vancomycin trough concentrations adequate for optimal dosing?. Antimicrob. Agents Chemother. 62 (1), e02042-17. 24165176 10.1128/AAC.01653-13 PMC3910745

[B14] Oda K. Jono H. Nosaka K. Saito H. (2020). Reduced nephrotoxicity with vancomycin TDM guided by AUC vs trough 15–20 μg/mL. Int. J. Antimicrob. Agents 56 (4), 106109. 10.1016/j.ijantimicag.2020.106109 32721597

[B15] Pai M. P. Neely M. Rodvold K. A. Lodise T. P. (2014). Innovative approaches to optimizing the delivery of vancomycin in individual patients. Adv. Drug Delivery Reviews 77, 50–57. 10.1016/j.addr.2014.05.016 24910345

[B16] Patel S. Preuss C. Bernice F. (2024). Vancomycin. In: Statpearls. Treasure Island (FL): StatPearls Publishing. 29083794

[B17] Pham J. T. (2020). Challenges of vancomycin dosing and therapeutic monitoring in neonates. J. Pediatr. Pharmacol. Ther. 25 (6), 476–484. 10.5863/1551-6776-25.6.476 32839651 PMC7439954

[B18] Regen R. B. Schuman S. S. Chhim R. F. Arnold S. R. Lee K. R. (2019). Vancomycin treatment failure in children with MRSA bacteremia. J. Pediatr. Pharmacol. Ther. 24 (4), 312–319. 10.5863/1551-6776-24.4.312 31337994 PMC6633277

[B19] Rybak M. J. Le J. Lodise T. P. Levine D. P. Bradley J. S. Liu C. (2020). Therapeutic monitoring of vancomycin for serious MRSA infections: revised consensus guideline. Am. J. Health Syst. Pharm. 77 (11), 835–864. 10.1093/ajhp/zxaa036 32191793

[B20] Schouwenburg S. Preijers T. Flint R. B. Wildschut E. D. Koch B. C. De Winter B. C. (2025). Prediction of vancomycin area under the curve with trough concentrations only: performance evaluation of pediatric population pharmacokinetic models. J. Infect. Dis., 231 (5), e882–e890. 10.1093/infdis/jiad487 39903655 PMC12128073

[B21] Siddiqui A. H. Koirala J. (2018). Methicillin-resistant staphylococcus aureus. In: Statpearls. Treasure Island (FL): StatPearls Publishing. 29489200

[B22] Sun C. Tan D. Yu J. Liu J. Shen D. Li S. (2023). Predictive models for sepsis in children with *S. aureus* bloodstream infections: a retrospective cohort study. BMC Pediatr. 23 (1), 496. 10.1186/s12887-023-04317-2 37784062 PMC10544563

[B23] Tkachuk S. Collins K. Ensom M. H. (2018). The relationship between vancomycin trough concentrations and AUC/MIC ratios in pediatric patients: a systematic review. Pediatr. Drugs 20 (2), 153–164. 10.1007/s40272-018-0282-4 29344778

[B24] Vali L. Jenkins D. R. Vaja R. Mulla H. (2021). Personalized dosing of vancomycin: a prospective and retrospective comparative quasi-experimental study. Br. J. Clin. Pharmacol. 87 (2), 506–515. 10.1111/bcp.14411 32495366

[B25] Woldu H. Guglielmo B. J. (2018). Incidence and risk factors for vancomycin nephrotoxicity in acutely ill pediatric patients. J. Pharm. Technol. 34 (1), 9–16. 10.1177/8755122517747088 34860973 PMC5998467

